# Analysis of Litigation Trends in Body Contouring Procedures Over the Last 45 Years

**DOI:** 10.1093/asjof/ojag138.006

**Published:** 2026-07-24

## Abstract

**Background:**

Body contouring procedures, including abdominoplasty and liposuction, are consistently among the most frequently performed cosmetic surgeries in the United States with approximately 170,000 abdominoplasties performed in 2024. Despite their popularity, these procedures carry significant risk for complications, including infection, seroma/hematoma formation and poor scarring. These adverse events, along with unsatisfactory cosmetic results, often serve as the basis for litigation. Additionally, while body contouring procedures fall under the scope of practice of plastic surgeons, they have also commonly been performed by non-plastic surgery physicians, such as general surgeons, OBGYN, bariatric surgeons, as well as OMFS and ENT. This project aims to examine the patterns behind body contouring procedure litigations and to compare the different basis of litigations, case outcomes, and complications amongst the different specialties

**Methods:**

The Westlaw legal database was queried for medical malpractice cases related to abdominoplasty and body contouring procedures from 1980 to 2025. Inclusion criteria included any litigation case where the defendant was a physician performing the specified procedures. Cases involving insurance companies, product manufacturers, or non-medical facilities were excluded. Cases were evaluated based on cause of action (malpractice, lack of informed consent, breach of contract, battery, gross negligence, and wrongful death), defendant specialty, verdict outcome, damages awarded and surgical complications.

**Results:**

A total of 172 cases were screened, with 114 meeting inclusion criteria. Most procedures were performed by plastic surgeons (n=86, 75.4%), followed by general surgeons (n=11, 9.6%) and OMFS (n=6, 5.2%). Plaintiffs were predominantly female (n=107, 93.8%) with a mean age of 44.4 years. Abdominoplasty with liposuction was the most common index procedure (n=64, 56.1%), followed by extremity liposuction (n=38, 33%) and panniculectomy (n=6, 5%). Nearly all cases involved claims of medical malpractice (n=98, 85.9%), with additional allegations including lack of informed consent (n=36, 31.5%), gross negligence (n=17, 14.9%), wrongful death (n=15, 13%), breach of contract (n=5, 4.3%), and battery (n=4, 3.5%).

Verdicts most often favored the surgeon (n=73, 64%). Among plaintiff verdicts (n=32, 28%) and settlements (n=9, 7.8%), nearly all resulted in financial awards, with a mean payout of $1,377,822. Four surgeons lost their medical licenses; three also received jail sentences. Of eight bowel perforations, six occurred during liposuction procedures (four by plastic surgeons, one by an OBGYN, and one by a general surgeon). Sepsis occurred in 10 cases and was the leading cause of wrongful death (n=4), followed by pulmonary embolism in seven cases (associated with three wrongful deaths), all occurring after abdominal liposuction.

**Conclusion:**

As the number of abdominoplasty and liposuction procedures continues to rise, associated litigation surrounding these procedures is also expected to increase. This study demonstrates that medical malpractice remains the leading basis for claims, with a substantial proportion also involving a lack of informed consent as well as wrongful death. These findings emphasize the need for thorough preoperative counseling as well as heightened vigilance for postoperative complications—such as sepsis and pulmonary embolism—which were major contributors to patient mortality.

